# Is It Possible to Mitigate Fear of Fireworks in Dogs? A Study on the Behavioural and Physiological Effects of a Psychoactive Supplement

**DOI:** 10.3390/ani14071025

**Published:** 2024-03-28

**Authors:** Daniela Ramos, Karina V. B. Yazbek, Amanda C. Brito, Barbara Georgetti, Luisa M. L. Dutra, Fabiola O. P. Leme, Angélica S. Vasconcellos

**Affiliations:** 1 Psicovet–Medicina Veterinária Comportamental, Jundiaí 13202-242, Brazil; barbarageorgetti@gmail.com; 2 Biolab Sanus Farmacêutica Ltda, São Paulo 04545-042, Brazil; kayazbek@yahoo.com.br (K.V.B.Y.); abrito@biolabfarma.com.br (A.C.B.); 3 Departamento de Ciências Biológicas, Pontifícia Universidade Católica de Minas Gerais, Belo Horizonte 30535-901, Brazil; lmldutra@sga.pucminas.br (L.M.L.D.); angelicavasconcellos@pucminas.br (A.S.V.); 4 Escola de Veterinária, Universidade Federal de Minas Gerais, Belo Horizonte 31270-901, Brazil; fabiolapaesleme@vetufmg.edu.br

**Keywords:** canine, noise, tryptophan, valerian, passiflora, cortisol, clinical animal behaviour

## Abstract

**Simple Summary:**

Fear of fireworks is one of the most common behavioural problems in dogs worldwide. From more static fearful behaviours such as freezing, hiding, and trembling to more active reactions such as aggression, running, barking, and even self-mutilation, many dogs suffer from firework fear but only a small proportion of their owners seek help from a specialist. As many owners prefer to use more natural products instead of medication for their dogs, we tested the efficacy of a supplement made of tryptophan, valerian, and passiflora on 44 dogs fearful of fireworks. Each of them received either the supplement or a placebo, plus environmental and behavioural recommendations to carry out during 2020 Christmas and 2021 New Year’s Eve when their behavioural (rated by the owners) and stress (measured via salivary cortisol dosages) reactions were evaluated. Improvement was significantly greater in dogs that received the supplement both in their behaviours and in their physiological responses. The studied supplement, in conjunction with simple behavioural advice, showed to be a good strategy for controlling firework fear in dogs.

**Abstract:**

Canine fear of fireworks is a common problem worldwide, with serious implications for the welfare of both dogs and their owners. Therapies for the problem are available, and herbal and nutraceutical agents are increasingly suggested by professionals; nonetheless, studies on their real efficacy in reducing firework fear are lacking. In a randomised, double-blinded, placebo-controlled study, 44 dogs (25 in the “supplement” group and 19 in the “placebo” group) completed a long-term continuous treatment with either a supplement made of tryptophan, valerian, and passiflora or a placebo, including two real exposures to fireworks (on 2020 Christmas and 2021 New Years’ Eve, after 42 and 48 days of treatment, respectively). Owners of both groups received the same general environmental management and food/toy offering recommendations for trying with their dogs on those nights. Behavioural (measured by LSSS—Lincoln Sound Sensitivity Scale and PANAS—Positive and Negative Activation scale, as rated by the owners) and stress (measured via salivary cortisol measures) reactions were evaluated. Significantly greater fear decrease (LSSS) was recorded in the “supplement” dogs, as compared to the “placebo” group. Cortisol dosages on New Year’s Eve (“noisy” night) were in line with behavioural results; “supplement” dogs showed a smaller increase in the stress response from 22:30 to 00:30 h on New Year’s Eve and a greater decrease in their stress response from 02:30 h to 10:30 h on New Year’s Day compared to “placebo” dogs. Smaller cortisol levels were also shown by “supplement” dogs than “placebo” dogs on a controlled “quiet night” (27th December). Owners’ rates on PANAS remained stable during the whole period of therapy for both groups. The evaluated supplement, a combination of tryptophan, valerian, and passiflora, showed satisfactory results and rare side effects when treating dogs fearful of fireworks.

## 1. Introduction

Fear of noises is a problem often seen by veterinary behaviourists in Brazil [1] and worldwide [2], with fireworks specifically triggering fear in a large part of the dog population [3,4]. Non-social fears, such as noise fear, can make the fearful dog more susceptible to diseases as well as decrease its life expectancy [5]. In addition to constituting a problem that generates suffering for affected dogs [4,6], it can also compromise the well-being of owners, who are commonly unable to properly manage the problem and grow frustrated [6], even though less than a third of them seek professional advice [7]. The negative impacts on human beings arising from living with a dog presenting behavioural disorders such as fears and phobias should not be underestimated [8].

In countries like Brazil, where the use of fireworks is very common throughout the year and only some municipalities have legislation regulating their use, noise fear constitutes a major problem for dogs and their owners. A study conducted in the surroundings of two football stadiums in Belo Horizonte, a southeastern region of Brazil [9], showed that most dogs that inhabited the neighbourhood of a football stadium exhibited increased levels of fear and anxiety behaviours during football matches; the closer to the stadiums the dogs lived, the more intense these behaviours were. Although, in addition to the sound of fireworks, screams and other noises from the crowds and vehicles around the stadiums are very common on these occasions and are likely to contribute to the fear exhibited by the dogs [9], the reported results point to a relevant public health problem.

There are several therapies available to control canine fear of fireworks, from isolated measures of environmental management (e.g., closing of windows and curtains, use of noise cancelling or a “safe haven” place to which the dog is previously conditioned) to more robust measures (e.g., desensitisation and counterconditioning through firework sounds artificially played [10,11]) and psychoactive agents, both allopathic and homeopathic, nutraceuticals, aromas, synthetic pheromones, and Bach Flowers. There is, however, little scientific evidence of the effectiveness of several of these strategies [12].

According to Riemer [12], who investigated the owners’ perception of the effects of different strategies to mitigate the problem, the use of medication (especially alprazolam and dexmedetomidine), relaxation techniques, and offering of play or valuable foods during exposure to fireworks seem to be the most effective measures to control the problem. For more severe cases, psychoactive agents are important and seem effective [12]. These can be used continuously from before and during the firework event, as in the case of imepitoin, or occasionally a few hours before, as with trazodone, dexmedetomidine, and gabapentin [13,14,15,16]. There is, however, a real resistance by the owners to the use of psychotropic drugs and a great interest in alternative therapies [17].

Agents considered by the owners as more natural, such as homeopathic or herbal products, aromas, Bach Flowers, nutraceuticals such as l-theanine and alpha casozepine, or even cannabidiol (CBD), are broadly available in the pet market. However, there is not sufficient efficacy evidence for all of them, with some rigorous studies presenting positive results of the agents, e.g., [18,19,20,21,22,23], but others failing in different aspects (e.g., uncontrolled studies, few participants, lack of effect, lack of behavioural responses analysis), e.g., [24,25,26,27,28,29,30], or demonstrating a placebo effect, e.g., [31].

Further controlled studies in dogs are therefore needed to uncover more of the real behavioural effects of these agents, such as tryptophan. This has been evaluated in terms of its physiological effects in sled dogs [32,33] and behavioural responses in laboratory dogs [34], client-owned compulsive [35], aggressive [36], and anxious dogs [37], but with very inconsistent results, and never evaluated in dogs fearful of fireworks.

## 2. Aims

This research aimed at evaluating the behavioural and physiological effects of a psychoactive supplement containing a nutraceutical (tryptophan) and two herbal agents (valerian and passiflora) to mitigate firework fear in fearful dogs submitted to natural exposure to fireworks during Christmas and New Year’s Eve. We hypothesised that the supplement would have an effect on dogs’ fear; in line with this hypothesis, we predicted that the supplement use would result in a lower frequency of fearful behaviours and better physiological stress parameters when treated dogs were compared to equally fearful dogs that received a placebo instead.

## 3. Materials and Methods

### 3.1. Recruitment

The survey was advertised on the researchers’ Instagram and Facebook pages as well as on WhatsApp groups of veterinarians. The advertisement, released during July and August 2020, looked for dogs with fear of fireworks, residents of the city of São Paulo and surroundings, whose owners were willing to implement behavioural therapy for their dogs for two months and who intended to spend Christmas and New Year’s Eve with them. Owners contacted the main researcher (DR) by email and, during those contacts, DR verified whether the dog and owner met the criteria for the study (below).

Inclusion criteria were as follows:

♣ Dogs had to be at least six months old (maximum 12 years old if dog was small/medium—<20 kg, and eight years old if large—≥20 kg);
♣ Dogs had to be living with the family for at least six months;
♣ Dogs had to be living in the current house for at least four months;
♣ Dogs had to accept to be medicated;
♣ Dogs had to exhibit firework fear at home and in the presence of owners;
♣ Owners had to agree to take the dogs to the laboratory for medical tests, and to treat them with an agent (psychoactive supplement or placebo) for two months;
♣ Owners had to agree to come to the behaviour clinic for two behavioural consultations and to fill out forms about the dog’s behaviour at home;
♣ Owners had to agree to carry out dog saliva collections at home, according to instructions and training received;

Exclusion criteria were as follows:

♣ Dogs so afraid of fireworks that they were never relaxed during the last weeks of the year;
♣ Dogs aggressive against people or involved in dog–dog aggression at home;
♣ Dogs taking any kind of psychoactive agents or pheromone therapy;
♣ Dogs having any physical illness;
♣ Dogs being pregnant/lactating (females) or used for breeding (males/females);
♣ Dogs living in kennels.

### 3.2. Initial Behavioural Consultations and Selection

During September and October 2020, behavioural consultations led by the first author (DR) with all the recruited dogs (one at a time) were carried out at a veterinary behaviour clinic (i.e., Psicovet Canine and Feline Behaviour Centre) located in São Paulo. During the consultations, dogs were weighed, and general demographic data of the dogs and their owners were collected. The owners were also provided with detailed information about the research, including the medical examinations that had to be performed on their dogs, to support the decision on their selection. For these, owners took their dogs to the laboratory where exams were carried out free of charge. The exams were as follows: complete blood count, renal function (serum creatinine and urea), liver function (serum ALT, AST, ALP), and thyroid profile (serum TSH, total and free T4). Only those dogs that presented results representing the norms for healthy dogs were selected for the research.

The following questionnaires were also filled out during initial behavioural consultations: Lincoln’s Sound Sensitivity Scale (LSSS-Initial—before the onset of treatment) and Positive and Negative Activation Scale (PANAS-Initial—before the onset of treatment). While the first questionnaire assesses the frequency and intensity of behaviours associated with fear of noise in dogs [10], the second evaluates the dogs’ temperament in terms of their responses to daily positive and negative stimuli—Positive (PA) and Negative (NA) Activations [38,39]. Given the complexity levels of the questionnaires, the researcher read the PANAS Scale questions and filled in the answers provided by the owners, whereas the LSSS Scale was filled in by the owners.

As part of the behavioural consultations, owners were also instructed on the collection of saliva from the dogs (which would be performed on 27th/28th December—“quiet night”—and 31st/01st January—“noisy night”) and received all the necessary materials for collection at home; this included a demonstration video. Collections were to be made in a friendly manner by introducing a cotton swab to the dog’s mouth and then keeping it there while making gentle movements for a few minutes until it was completely wet. Owners were also instructed to habituate their dogs to the collection procedure weeks in advance by just doing it occasionally and in a friendly manner, such that on the collection nights, dogs would nicely accept the procedure. Saliva samples were to be stored in appropriate tubes (provided along with the cotton swabs) and kept in domestic freezers from immediately after collection until the owners brought them to the final behavioural consultation in January 2021.

Owners also received general guidance on how to act with their dogs on Christmas and New Year’s Eve (i.e., “Task Force Against Fireworks” in Supplementary Materials instructions prepared by the authors for the study). This included safety aspects such as the use of a dog collar with identification, the closing of windows and doors, and keeping close to the dog at all times. It also suggested exercising the dog a couple of hours before the expected fireworks display, taking the dog to a calm room in the house where fireworks would not be so loud, and offering delicious food or exciting toys when fireworks were on, if the dog accepted them.

After the dogs’ selection was confirmed upon their results on the laboratory tests representing the norms for healthy dogs, the vials containing the pills (supplement or placebo, in unidentifiable bottles) were delivered to the owners in their homes.

### 3.3. Treatment Starting on 15th November

Half of the dogs were randomly allocated to the “supplement” group, while the other half went to the “placebo” group. Neither the researchers nor the owners knew which agent each dog received. Treatment with supplement or placebo consisted of two daily doses (the first in the morning and the second at night, with a twelve-hour interval) from 15 November 2020 to 1 January 2021 (i.e., 48 days of treatment). The supplement named QUETIN^®^ contained three psychoactive ingredients—the nutraceutical tryptophan and two herbal agents (valerian and passiflora). The average dosages of each ingredient received by the dogs were tryptophan (10.4–15.6 mg/kg twice a day), valerian (2.1–3.1 mg/kg twice a day), and passiflora (10–12.5 mg/kg twice a day)—plus inactive ingredients. The placebo was composed of the same inactive ingredients present in the supplement.

During the treatment period, owners were contacted by telephone on several occasions to be reminded of questionnaires to be answered or sample collections to be made, and could also contact the researcher if they wished to. During these contacts, they could report their dogs’ reactions to the treatment as well as ask any questions they had.

### 3.4. First Official Contact (after 35 Days of Treatment)

On 20 December 2020, all owners were contacted for an initial behavioural evaluation after 35 days of therapy. They were asked about their initial perceptions on their dogs’ fearful behaviours, about the occurrence of side effects, and any improvement in general behaviour during that period. It was assumed that dogs were likely exposed to the noise of fireworks now and then given that São Paulo is a region where people often set off fireworks on several occasions every week, so they would be able to assess their dogs’ current fear of noise.

### 3.5. Second Official Contact (after Christmas, 42 Days of Treatment)

On the morning of 27 December 2020, all owners were contacted and asked to respond to the Lincoln Sound Sensitivity Scale (LSSS-Christmas—after 42 days of treatment) regarding the fear behaviour observed in their dogs during the Christmas period (fireworks are very common around this time in Brazil). They were also asked about the occurrence of side effects during that period.

They were also reminded about the saliva collections that were to be carried out on that “quiet night”, including the following morning (27th/28th December) at the following times: 22:30 h, 00:30 h, 02:30 h, and 10:30 h. In case there was any kind of loud noise (i.e., from fireworks, thunderstorm, crowds) on that day or night, or if it was an unusual day, owners were instructed not to collect the samples on that night but to do it on one of the following nights (28–30 December), as long as there was not any loud noise and it was a typical day. The salivary cortisol values obtained from these samples (i.e., “quiet night”) would serve as a basis for comparison with those obtained from samples collected on New Year’s Eve (“noisy night”) at the same four times.

### 3.6. Third Official Contact (on New Year’s Eve)

On the morning of 31 December 2020, all owners were contacted to be reminded of the saliva collections that were to be carried out on that “noisy night”, including the following morning (31st December/1st January) at the following times: 22:30 h, 00:30 h, 02:30 h, and 10:30 h. They were instructed to be next to their dogs at all times and follow the “Task Force Against Fireworks” instructions as much as possible. The main researcher kept in contact with all owners on that night via WhatsApp and telephone, reminding them about the collection times as well as clarifying any questions they had at any time during this period.

### 3.7. Fourth Official Contact (after New Year’s Eve, 48 Days of Treatment)

On 02nd January, all owners were contacted by telephone and questioned about (I) their perceptions of their dogs’ fearful behaviours during that period, (II) satisfaction with the therapy, and (III) intentions of future use of the agent. Owners were also reminded to stop giving the pills to the dogs, and the final behaviour consultations were scheduled. The occurrence of side effects was also questioned.

### 3.8. Final Behavioural Consultations

During January 2021, final behavioural consultations by DR with all dogs, one at a time, were performed at the same behaviour clinic in São Paulo in which the initial behavioural consultations took place. Owners completed the LSSS-Final (after 48 days of treatment) and the PANAS-Final (after 48 days of treatment). The researcher asked the owners about their final perceptions on their dogs’ general and fearful behaviours. The frozen saliva samples were brought by the owners and immediately transferred to the freezer at the clinic. Owners were informed which agent, placebo or supplement, their dogs received.

### 3.9. Salivary Cortisol

Salivary cortisol measurements were performed at Minas Gerais Federal University (UFMG) during March 2021. Immunoenzymatic methods (ELISA) were used, following the protocol of a commercial kit (Salimetrics^®^, Carlsbad, CA, USA), which had already been shown to be valid for measuring canine salivary cortisol [40].

### 3.10. Data Analysis

We ran General Linear Models (GLMs) to evaluate possible effects of the demographic profiles of the dogs (sex, age, breed, weight, presence/absence of other dogs in the household, type of household) on their allocation in the “supplement” or “placebo” groups (i.e., whether dogs’ profiles were different between groups), and possible effects of their demographic profiles and treatment (allocation between groups) on the owners’ answers to the questionnaires, and the questions about their perceptions regarding the treatment. The variables used in these models are described in Table 1. Then, Generalised Mixed Models (GLMMs; repeated measures) were run to evaluate the effects of the variables: treatment (supplement, placebo), sample (1—“quiet night” 22:30 h, 2—“quiet night” 00:30 h, 3—“quiet night” 02:30 h, 4—“quiet night” 10:30 h, 5—“noisy night” 22:30 h, 6—“noisy night” 00:30 h, 7—“noisy night” 02:30 h, 8—“noisy night” 10:30 h), night (quiet night, noisy night), and their interactions on dogs’ cortisol concentrations. Minimal adequate models were obtained by using the iterative method. We used “lme4”, “MASS”, and “car” packages to fit GLM/GLMM models in the R statistical software, version 3.5.2 (R, 2015).

Apart from these tests, we used Friedman and Mann–Whitney tests to check for the random distribution of the missing saliva samples.

All results were analysed based on statistical significance (α ≤ 0.05).

## 4. Results

### 4.1. Participants

A total of 150 owners initially contacted the main researcher showing interest in taking part in the study. After being given detailed information, and as inclusion and exclusion criteria were verified, 83 confirmed their interest, and behavioural consultations for their dogs were scheduled. Sixty-three of them came to the consultations (some reported having thought better and decided not to participate, others declared the dogs were sick, and others simply did not show up for the consultation). Medical examinations were performed on these 63 dogs, with 47 having results representing the norms for healthy dogs, thus being selected for the research. Three dogs were later excluded during treatment due to the following reasons: an unneutered female became pregnant (“placebo” group), one female had seizures (“placebo” group), and another female had Ehrlichiosis (“supplement” group). Thus, 44 dogs completed the treatment, 25 in the “supplement” group and 19 in the “placebo” group. As shown in Table 2, dogs in the “placebo” and “supplement” groups did not differ in terms of their demographic characteristics nor in their noise fear or personality profiles, the latter measured via LSSS and PANAS, respectively.

### 4.2. Treatment

The number of responses to the questionnaires (PANAS and LSSS) and to the exploratory questions per group (“placebo” and “supplement”) recorded at each collection moment (35, 42, 48 days, and at the final consultation) can be seen in Table 3.

#### 4.2.1. Initial Perception (after 35 Days of Treatment and after Christmas)

During the first official contact, the majority of owners reported having heard fireworks during the initial 35 days of treatment (i.e., 17 in the “supplement” group and 11 in the “placebo” group; estimate = −0.028; *p* = 0.684), but overall there was little improvement in their dogs’ fireworks fear (i.e., ten “no change” and seven “better” in the “supplement” group versus seven “no change” and four “better” in the “placebo” group; estimate = 0.2203, *p* = 0.783). Side effects were poorly reported in both groups (i.e., two sleepy dogs, one dog with decreased appetite, and one vomiting dog in the “supplement” group versus one sleepy dog, one dog with decreased appetite, and one dog with diarrhoea in the “placebo” group). Eleven owners in the “supplement” group and five in the “placebo” group reported that overall, their dogs behaved better during treatment (estimate = −0.186; *p* = 0.509).

Regarding the second official contact (i.e., after 42 days of treatment), in both groups, owners declared that they had heard fewer fireworks on Christmas Eve and Day compared to the previous year; nine owners in each group stated that they had not heard firework noise during Christmas and therefore did not respond to the LSSS-Christmas. Considering the remaining ones, in both groups, there was a decrease in the noise fear when LSSS-Christmas was compared to LSSS-Initial. The decrease seen in the “supplement” group was significantly greater (33% “supplement” versus 23% “placebo”; estimate = 6.396, *p* < 0.001).

#### 4.2.2. Final Perceptions (After 48 Days of Treatment and at Final Consultations)

During the fourth official contact, all owners confirmed they heard fireworks during New Year’s Eve and Day, thus being able to observe well their dogs’ fear response. Most owners in both groups rated their dogs as behaving better when exposed to fireworks this year (i.e., twelve “better”, seven “much better”, five “no change”, and one “worse” in the “supplement” group versus six “better”, two “much better”, ten “no change”, and one “worse in the “placebo” group; estimate = 0.3, *p* = 0.261). In both groups, most owners declared themselves to be satisfied with the therapy (i.e., thirteen “satisfied”, nine “neither satisfied nor unsatisfied”, and three “unsatisfied” in the “supplement” group versus nine “satisfied”, eight “neither satisfied nor unsatisfied”, and two “unsatisfied” in the “placebo” group; estimate = 0.143, *p* = 0.557). Most of them also stated that they would use the agent again in the future (fifteen “would use”, eight “would not use”, and two “don’t know” in the “supplement” group and fourteen “would use”, five “would not use” in the “placebo” group; estimate = −0.078, *p* = 0.779).

During final behavioural consultations at the clinic, most owners reported their dogs’ fear of fireworks as better after the end of therapy (i.e., eight “better”, nine “much better”, five “no change”, and three “worse” in the “supplement” group versus six “better”, four “much better”, and nine “no change” in the “placebo” group; estimate = −0.205, *p* = 0.341). Improvements in their dogs’ general behaviour, such as less reactivity, less fear of thunderstorms, more sociability with other dogs, more relaxation, and gentleness with owners, were reported by twelve owners in the “supplement” group and seven owners in the “placebo” group; there were no significant differences between groups in this regard (estimate = 0.107, *p* = 0.678). All owners reported having applied most instructions described in the “Task Force Against Fireworks”. Given the option to rate from 0 to 10 (0 being none and 10 all of it) how much of the “Task Force Against Fireworks” they had put into practice during New Year’s Eve, owners in the “supplement” group rated 7.92 on average, whilst owners in the “placebo” group rated 7.81 (estimate = 0.012, *p* = 0.915).

As for the LSSS-Final scale compared to LSSS-Initial, there were differences between the groups; dogs in the “supplement” group showed a significantly greater decrease in their noise fear (41% “supplement” versus 27% “placebo”; estimate = 0.219, *p* < 0.001). Regarding the PANAS-Final scale compared to PANAS-Initial, the same result was observed in both “supplement” and “placebo” groups; both positive and negative activation remained stable after therapy (estimate = 0.016, *p* = 0.107 and estimate= 0.01, *p* = 0.277, respectively).

#### 4.2.3. Salivary Cortisol

All owners performed the eight planned salivary collections. However, even having been instructed on how to collect dog saliva, having all previously received a video demonstrating the procedure, and practiced it to habituate their dogs, some of them had difficulties obtaining saliva, with samples resulting in an insufficient amount of saliva. Out of a total of 352 salivary samples (i.e., 8 samples from each of the 44 dogs), 113 of them (32%) had insufficient amounts of saliva and therefore were excluded from cortisol dosages. As excluded samples were randomly distributed between groups and along collection times (*p* > 0.05 for both), we could proceed with dosage and statistical analysis of the remaining ones.

**Figure 1:**
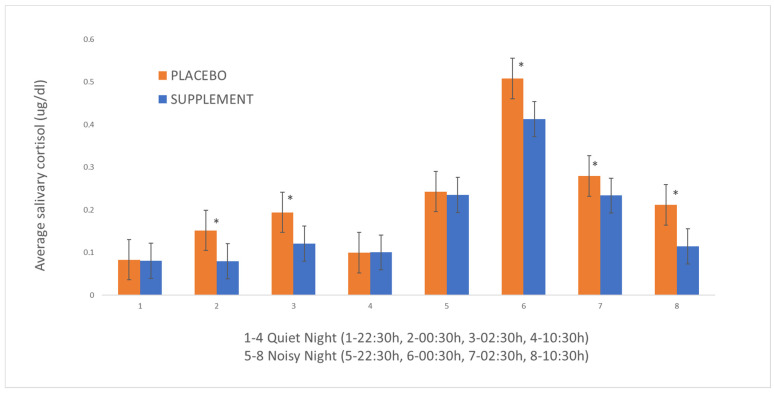
Mean salivary cortisol concentrations (µg/dL) collected from the dogs from the groups “supplement” and “placebo” throughout a “quiet night” (1–4) and a “noisy night” (5–8) for a study on the effects of a psychoactive supplement to mitigate the fear of fireworks. Asterisks indicate statistical differences between groups (“supplement” versus “placebo”).

Figure 1 shows the distribution of mean salivary cortisol values for the four samples collected on the “quiet night” (i.e., 27th/28th December 1—22:30 h, 2—00:30 h, 3—02:30 h, and 4—10:30 h) and on the “noisy night” (i.e., 31st December/01st January—5—22:30 h, 6—00:30 h, 7—02:30 h, and 8—10:30 h) for both groups. The statistical analysis showed an interaction effect of sample order (1–8) with the variable “treatment” (estimate = 0.036, *p* < 0.001) on cortisol concentrations, that is, cortisol values differed between “placebo” and “supplement” dogs depending on the collection hour. There was also an interaction between treatment and the night (“noisy night” versus “quiet night”); saliva cortisol values were significantly higher in the “placebo” group in two moments of the “quiet night”, compared to three moments of the “noisy night” (estimate = 0.147, *p* < 0.001).

Analysing what we call “midnight peak” (i.e., the increase in cortisol levels between 22:30 and 00:30 on New Year’s Eve), there was a statistical difference between the groups (estimate = 0.638, *p* < 0.001), with a significant greater increase in dogs that received the placebo. Similarly, analysing what we call the “ten-thirty fall” (i.e., the decrease in cortisol levels between 00:30 h and 10:30 h on New Year’s Eve) there was also a statistical difference between the groups (estimate = 0.685, *p* < 0.001), with a greater decline in dogs that received the supplement.

When each set of the samples (1–8) was analysed independently, and considering their median values, higher values for the “placebo” group were verified in samples 2 and 3 (“quiet night”; 00:30 h: estimate = 0.649, *p* < 0.001 and 02:30 h: estimate = 0.477, *p* < 0.001) and 6, 7, and 8 (“noisy night”; 00:30 h: estimate = 0.208, *p* < 0.001, 02:30 h: estimate = 0.17, *p* < 0.001 and 10:30 h; estimate = 0.615, *p* < 0.001).

## 5. Discussion

In this study, behavioural and stress reactions of firework-fearful dogs were evaluated, in response to a supplement made of tryptophan, valerian, and passiflora, or a placebo. In participant dogs receiving either the supplement or the placebo, fear responses to fireworks decreased, and their general behaviour improved during treatment. The obtained results were satisfying for the owners, as most of them declared themselves satisfied and willing to use the therapy again in the future. Most important, however, is that dogs in the “supplement” group had significantly greater declines in their fear responses to fireworks than dogs in the “placebo” group on both Christmas and New Year’s Eve (after Christmas: 33% versus 23% improvement on LSSS; after New Year’s Eve: 41% versus 27% improvement on LSSS). Salivary cortisol dosages from the dogs followed the behaviour results, thus pointing in the same direction. During New Year’s Eve, dogs in the “placebo” group had a greater increase in cortisol levels between 22:30 h and 00:30 h (“midnight peak”), which is the interval in which firework activity reaches its peak. In addition to that, dogs in the “supplement” group had a greater reduction in their cortisol levels between 00:30 h and 10:30 h (“ten-thirty fall”), which is the interval in which firework activity notably decreases. At 22:30 h on New Year’s Eve, both groups had similar levels of stress and were already under the effects of the fireworks that likely started earlier. “Supplement” dogs at 10:30 h on New Year’s Day reached cortisol levels much closer to what they had shown on a quiet morning, which is an impressive result, as some dogs may take more than a day to recover from a firework event [4]. Administration of the supplement resulted in a decreased reactivity and a more rapid recovery of their stress response.

Salivary cortisol measures coming from careful and precise collections, processing, and analyses can be a reliable non-invasive acute stress marker in dogs [40,41,42,43]. In a previous study, such measures were efficiently used to measure the stress response of dogs exposed to a thunderstorm recording in a home setting, but many owners declared that it was difficult to collect enough saliva, or their dogs resisted it [6]; the same situation also took place in our study. Nevertheless, taken together, our behavioural and physiological results point to acceptable evidence that dogs in the “supplement” group exhibited lower stress responses, even though they had been exposed to similar stressors. The studied supplement seems to have decreased the dogs’ fear of noise, not simply by inhibiting behavioural responses, but also by alleviating physiological stress reactions, thus leading to less fearful reactions.

The effect of the supplement on the stress response of the participant dogs went beyond the firework exposure. Dogs in the “supplement” group showed smaller salivary cortisol levels than dogs in the “placebo” group during the “quiet night”. Since the week between Christmas and New Year constitutes a festive period and there were rainy days/nights around this time, it might be that stressors present on the “quiet night” were mild noises. Alternatively, cortisol increase on the “quiet night” might be due to the collection procedure per se. In any case, the supplement also acted by somehow mitigating the stress response arising from minor disturbing stimuli present even on an apparently calm night.

Improvement in the placebo group was not unexpected, given the “placebo effect” on the owners’ part, which can be very high in behavioural studies, as also noted by [31]. Furthermore, the “Task Force Against Fireworks”, which owners followed to a high degree, likely contributed to their dogs’ improved behaviours during Christmas and New Year’s Eve. According to Riemer [12], even less robust recommendations such as environmental modifications and ad hoc counterconditioning using valuable items, as included in our instructions, can be very effective in decreasing fear of fireworks. We should mention, however, that 40 owners declared they had already followed advice similar to the ones contained in our “Task Force Against Fireworks” (e.g., shut windows and curtains, take the dog to a safe and calm place within the house, offer treats) in previous years and had not achieved success. Although they declared to have followed the provided recommendations during the study, as in the case of offering the most delicious foods and interesting play, 10 owners did not offer them, and 13 dogs refused to accept them. Taking into consideration the fact that many owners reported that they had perceived fewer fireworks compared to previous years, and that this might be due to the pandemic period in which this study was carried out, this improvement can be attributed partly to a lower number of fireworks displayed on Christmas 2020 and New Year’s Eve 2021. The placebo effect, “Task force against fireworks”, and fewer fireworks might have led to less fearful behaviours in the “placebo” dogs, but the addition of a psychoactive supplement pushed improvement further in the “supplement” group.

Neither dogs in the “supplement” group nor the “placebo” group showed improvement in their positive and negative activations in PANAS, which is a validated reliable tool for measuring sensitivity to reward and aversive stimuli [38,39]. The fact that participants detected improvement in their dogs’ general behaviours in addition to the alleviation of noise fear might be seen as a paradox. A possible interpretation of this discrepancy is that the period of forty-eight days of therapy, even if it is considered successful in reducing the fear of the tested dogs, was still insufficient to lead to changes in their positive and negative activations, as measured by the PANAS questionnaire. As these dogs had experienced noise fear for several years, the continuation of such therapy with several less stressful exposures to fireworks may lead to more improvement, including changes to their sensitivity to other stimuli.

After an initial 35-day treatment, when simply asked, most owners had not seen great changes in their dogs’ fear responses to fireworks. Despite this, a week later (i.e., after 42 days of treatment), LSSS-Christmas revealed a greater improvement in the “supplement” group, compared to the “placebo” group. It is possible that, as a more detailed and objective investigation, LSSS would have also detected improvement at 35 days if it had been applied on the first contact instead of the one subjective question on how they had perceived the progression of their dogs’ fear of noises during that initial period. Alternatively, it is also possible that the effects of the supplement became noticeable only after 42 days of treatment. Indeed, since tryptophan—unlike valerian and passiflora but like other serotoninergic agents—may need weeks to exert clinical effects, such improvement seen in the participant dogs from 42 days of therapy on might be either due to tryptophan alone or tryptophan potentiated by valerian and (or) passiflora.

In humans, research has shown that tryptophan can be a determinant in mood, cognition, and behaviour, and results of clinical trials have shown its efficacy for moderate psychiatric disorders; for a review, see [44]. In veterinary behaviour medicine, tryptophan has shown inconsistent results [32,33,34,35,36,37] and studies on valerian and passiflora are lacking. The studied supplement, a combination of tryptophan, valerian, and passiflora, has shown evidence of satisfactory results after 42 days of regular daily use, with a further decline in the dogs’ fear of noise after 48 days of use. Further therapy using this supplement should start at least one-and-a-half months ahead of the fearful noisy event, and ideally in combination with environmental management and ad hoc counterconditioning using valuable items.

## 6. Conclusions

The studied supplement, a combination of tryptophan, valerian, and passiflora, was found to induce both behavioural and physiological correlates of fear reduction (with relatively infrequent side effects) when used to treat dogs suffering from fear of fireworks. For maximum results, this pharmacological treatment should be combined with behavioural recommendations, such as environmental modifications and ad hoc counterconditioning using valuable items.

## Figures and Tables

**Table 1:** Response variables of General Linear Models run to evaluate possible effects of the demographic profiles of the dogs (sex, age, breed, weight, presence/absence of other dogs in the household, type of household) on their allocation in the “supplement” and “placebo” groups (Treatment), and possible effects of their profiles and treatment on the owners’ answers to the questionnaires.

Response Variables	Description
Treatment	Compound Placebo
Lincoln Sound Sensitivity Scale (LSSS)	LSSS-Initial ^1^ LSSS-Christmas ^2^ LSSS-Final ^3^ LSSS-Christmas/LSSS-Initial ^4^ LSSS-Final/LSSS-Initial ^5^
Positive and Negative Activation Scale (PANAS)	PA-Initial ^6^ PA-Final ^7^ PA-Final/PA-Initial ^8^ NA-Initial ^9^ NA-Final ^10^ NA-Final/NA-Initial ^11^
Perception of fearful behaviours	After 35, 48 days of treatment, and at the final consultation: better, much better, no changes, worse, much worse
Improvement in general behaviours	After 35 days of treatment, and at the final consultation: yes, no, don’t know
Satisfaction with the therapy	After 48 days of treatment: satisfied, not satisfied nor unsatisfied, unsatisfied
Compliance to the Task Force Against Fireworks	At the final consultation: rate from 0 to 10 (0 being none and 10 all of it) regarding how much of the “Task Force Against Fireworks” they had put into practice during New Year’s Eve
Future use of the agent	After 48 days of therapy: would use, don’t know, wouldn’t use

^1^ Lincoln Sound Sensitivity Scale answered before onset of treatment (September/October 2020). ^2^ Lincoln Sound Sensitivity Scale answered after 42 days of treatment (27 December 2020). ^3^ Lincoln Sound Sensitivity Scale answered after 48 days of treatment (January 2021). ^4^ To obtain LSSS-Christmas/LSSS-Initial, the marks attributed to LSSS-Christmas were divided by the marks of LSSS-Initial. ^5^ To obtain LSSS-Final/LSSS-Initial, the marks attributed to LSSS-Final were divided by the marks of LSSS-Initial. ^6^ Positive Activation Scale answered before the onset of treatment (September/October 2020). ^7^ Positive Activation Scale answered after 48 days of treatment (January 2021). ^8^ To obtain PA-Final/PA-Initial, the marks attributed to PA-Final were divided by the marks of PA-Initial. ^9^ Negative Activation Scale answered before the onset of treatment (September/October 2020). ^10^ Negative Activation Scale answered after 48 days of treatment (January 2021). ^11^ To obtain NA-Final/NA-Initial, the marks attributed to NA-Final were divided by the marks of NA-Initial.

**Table 2:** Demographic and behavioural data of the dogs allocated to the “Supplement” (*n* = 25) and the “Placebo” groups (*n* = 19) in a study on the effects of a psychoactive supplement on dogs fearful of fireworks.

		“SUPPLEMENT” GROUP	“PLACEBO” GROUP
DEMOGRAPHICAL DATA	Sex	40% Male 60% Female	52.6% Male 47.4% Female (estimate = −0.108, *p* = 0.677 *)
	Breed	68% cross-bred 32% pure-bred	47.4% cross-bred 52.6% pure-bred (estimate = −0.144, *p* = 0.569 *)
	Age	Average: 5.6 years	Average: 5.4 years (estimate = −0.003, *p* = 0.948 *)
	Neutering	24 Neutered, 1 intact	18 neutered, 1 intact (estimate = 0.497, *p* = 0.736 *)
	Household	72% house 28% flat	57.9% house 42.1% flat (estimate = 0.112, *p* = 0.676 *)
	Living with other dog (s)	64% yes 36% no	63.16% yes 36.84% no (estimate = 0.041, *p* = 0.878 *)
	Weight	Average: 16.52 kg	Average: 21.54 kg (estimate = 0.01, *p* = 0.442 *)
BEHAVIOURAL DATA	LSSS Initial	- Global Score GS (0–270): 106.6	- Global Score GS (0–270): 113.53 (estimate = 0.005, *p* = 0.456 *)
	PANAS Initial	PA+ (0–1): 0.64 NA− (0–1): 0.63	PA+ (0–1): 0.78 (estimate = 0.812 *p* = 0.274 *) NA− (0–1): 0.60 (estimate = −0.301 *p* = 0.747 *)

* Statistical values of the General Linear Models.

**Table 3:** The number of responses to the exploratory questions recorded in each interview (at 35, 42, and 48 days from the beginning, and after the end of the treatment) from owners of dogs allocated to the “Supplement” (*n* = 25) and the “Placebo” groups (*n* = 19) in a study on the effects of a psychoactive supplement on dogs fearful of fireworks.

35 Days			42 Days			48 Days			Final		
	Suppl. Group	Plac. Group		Suppl. Group	Plac. Group		Suppl. Group	Plac. Group		Suppl. Group	Plac. Group
Fireworks heard			Fireworks heard			Fireworks heard			Question not asked **		
No	8	8	No	9	9	No	0	0			
Yes	17	11	Yes	16	10	Yes	25	19			
General fear *			Question not asked **			General fear			General fear		
Much worse	0	0				Much worse	0	0	Much worse	0	0
Worse	0	0				Worse	1	1	Worse	3	0
No change	10	7				No change	5	10	No change	5	9
Better	7	4				Better	12	6	Better	8	6
Much better	0	0				Much better	7	2	Much better	9	4
Question not asked **			Question not asked **			Satisfaction with treatment			Question not asked **		
						Satisfied	13	9			
						Not satisfied nor unsatisfied	9	8			
						Unsatisfied	3	2			
Side-effect occurrence			Side-effect occurrence			Side-effect occurrence			Question not asked **		
Somnolence	2	1	Somnolence	0	0	Somnolence	0	0			
Reduced appetite	1	1	Reduced appetite	0	0	Reduced appetite	0	0			
Vomit/ diarrhoea	1	1	Vomit/ diarrhoea	0	0	Vomit/ diarrhoea	0	0			
Question not asked **			Question not asked **			Future use			Question not asked **		
						Would not use	8	5			
						Don’t know	2				
						Would use	15	14			
Question not asked **			Question not asked **			Question not asked **			Compliance to the Task Force		
									Mean rating	7.92	7.81
Improvement in General Behaviours			Question not asked **			Question not asked **			Improvement in General Behaviours		
No	14	14							No	13	12
Yes	11	5							Yes	12	7
Question not asked **			Fear reduction (according to LSSS)			Question not asked **			Fear reduction (according to LSSS)		
			% improvement	33%	23%				% improvement	41%	27%
Question not asked **			Question not asked **			Question not asked **			Positive and Negative activations (according to PANAS)		
									PA and NA have not changed		PA and NA have not changed

* This question was answered only by those who had heard fireworks from the beginning of the treatment up to that day. ** This question was not asked at this moment.

## Data Availability

The data presented in this study are available on request from the corresponding author. The data are not publicly available because they contain information that could compromise the privacy of research participants.

## Supplementary Materials

The following supporting information can be downloaded at: https://www.mdpi.com/article/10.3390/ani14071025/s1, The Task force against fireworks.
