# Supplementary material for: Is It Possible to Mitigate Fear of Fireworks in Dogs? A Study on the Behavioural and Physiological Effects of a Psychoactive Supplement
Source: Animals (Basel). 2024 Mar 28;14(7):1025. doi: 10.3390/ani14071025 (PMC11010855; doi:10.3390/ani14071025)
Supplement: Supplementary file 1 [file animals-14-01025-s001.zip › animals-2904313-supplementary.pdf]

TASK FORCE AGAINST FIREWORKS

Are you worried about the fireworks?

Let the TASK FORCE be with you!

1- Safety is essential. Close doors and gates and be very careful with people coming and going. Identification tags placed on dogs!

2 - Comfortable environment. Windows and curtains closed, and familiar ambient sound. Fans and air conditioner on so no one “dies” from the heat.

3 - Always be together, never leave the dog alone at home. Don't feel embarrassed about spending New Year's Eve supporting your little friend. Invite family and friends to celebrate at your home and combine celebration and TASK FORCE!

4 - Create a safe haven for your dog. It can be a bedroom, a corner of the living room or kitchen - as long as it muffles the sounds better, or even a bed or carrier that the dog is used to. In moments of greater fear, allow the dog to go to the safe haven and just be nearby to monitor.

5 - A vigorous activity such as a long walk or an energetic game is great option for the afternoon, as they can help the dog relax during the night.

6 - Prepare the feast! High-value foods such as special dry snacks, soft foods, and irresistible chew bones, including frozen ones, will be our trump card in this Task Force. Keep them prepared and offer them to the dog during noisy moments. If he/she doesn't want it, that's okay, save it and try to offer it later.

7 - Prepare the fun! Cool, special toys, the ones they love most, will also be an asset to us! Keep them separate and offer them during noisy moments. If the dog doesn't want them, try later.

8 - If your dog doesn't want to play or eat but appreciates a good massage or any other relaxing physical contact, as long as he/she is already used to it, do it. It is very important that he/she feels accompanied and welcomed.

Happy 2021!
